# Single Stage Simultaneous Core Decompression for Ficat Stage I and II Bilateral Femoral Head Osteonecrosis among Hip Surgeries done in a Tertiary Care Centre: A Descriptive Cross-sectional Study

**DOI:** 10.31729/jnma.6383

**Published:** 2021-04-30

**Authors:** Rishi Bisht, Dipesh Pariyar, Pramod Joshi

**Affiliations:** 1 Department of Orthopaedics, National Trauma Centre, National Academy of Medical Sciences, Kathmandu, Nepal

**Keywords:** *decompression*, *femur head necrosis*, *hip*, *osteonecrosis*

## Abstract

**Introduction:**

Osteonecrosis of femoral head is a disease affecting young individuals and often occurs in bilateral hips. The purpose of this study is to find out the prevalence of single stage simultaneous core decompression for Ficat stage I and II bilateral femoral head osteonecrosis among total hip surgeries done in a tertiary care center.

**Methods:**

A descriptive cross-sectional study was conducted at a tertiary care trauma centre from April 2017 and March 2020. Ethical approval was obtained from the Institutional Review Committee prior to data collection (Reference no: 673/2077/78). Convenience sampling was done. Patients undergoing hip surgeries were included in the study. Patients with missing data were excluded. Patients operated upto Ficat Stage IIb femoral head osteonecrosis were followed up. Data was analysed using the Statistical Package for Social Sciences version 22.0. Point estimate at 95% Confidence Interval was calculated along with frequency and percentage for binary data.

**Results:**

Out of 916 patients who underwent hip surgeries, 12 (1.31%) (0.57-2.04 at 95% Confidence Interval) underwent single stage simultaneous core decompression. Idiopathic cause in 6 (50%) and prolonged corticosteroid usage in 4 (33.33%) were the leading causes. Twelve (50%) of the treated hips were in Ficat stage IIa. At the end of one year, of the 11 patients who followed up, 12 (54.5%) hips had a good Harris hip outcome.

**Conclusions:**

Our study showed that a small proportion of hip surgeries were single stage simultaneous bilateral core decompression for osteonecrosis of femoral head in our setting. Our findings were similar to studies done internationally with a very low complication rate.

## INTRODUCTION

Osteonecrosis (ON) of the hip is a disease affecting young individuals of thirties and forties in which the living elements of bone in the femoral head die with the disruption of blood supply.^1,2^ Early surgical treatment, before the collapse of head yields the best result.^3^ Previous studies have described that osteonecrosis of femoral head (ONFH) often occurred in bilateral hips and the incidence was as high as 78%.^4^

It is advisable to treat both hips simultaneously rather than delaying the second procedure by weeks or months. It is a known fact that core decompression can postpone other reconstructive procedures like total hip arthroplasty (THA).^5^

The purpose of this study is to find out the prevalence of single stage simultaneous core decompression for Ficat stage I and II bilateral femoral head osteonecrosis among total hip surgeries done in our centre.

## METHODS

A descriptive cross-sectional study was conducted in the department of orthopaedics at National Trauma Centre between the period of April 2017 to March 2020. Ethical approval was obtained from the Institutional Review Board of National Academy of Medical Sciences prior to data collection (Reference no: 673/2077/78). Patients undergoing hip surgeries were included in the study. All patients with bilateral ONFH Ficat stage I, IIa and IIb were followed up for the study. Surgeries carried out by the same surgical team were included. Those with prior collapse of femoral head were excluded from the study along with hip diseases with local site infection. In addition, any patient with missing data was excluded from the study. Convenient sampling method was used. Sample size was calculated using formula,

$$\begin{array}{ll} \text{n} & ={\text{Z}}^{\text{2}}\times \text{p}\times \text{(1}-\text{p)}/{\text{e}}^{\text{2}}\\ & =1.96^{2}\times 0.5\times 0.5/0.05^{\text{2}}\\ & =384.16 \end{array}$$

where,

n = minimum required sample size
Z = 1.96 at 95% Confidence Interval (CI)
p = prevalence taken as 50% for maximum sample size
e = margin of error, 5%.

The required sample size was 384.16. As we used convenience sampling, we doubled the sample size to 768.32. By adding a 10% non-response rate, a final sample size of 846 was obtained. But we included 916 patients in the study.

Out of the total sample size, the contact details of patients who underwent single stage simultaneous bilateral core decompression were retrieved from the hospital record section and following their consent regarding the study all previous relevant data during follow ups were recorded. Clinical and radiographic examination conducted during follow ups at one month, three months, six months and one-year postsurgery were recorded. ONFH was classified according to Modified Ficat and Arlet Classification. Safety of the procedure was ascertained with presence or absence of perioperative complications like fracture along the core track, perforations of femoral head, infections and deep vein thrombosis. Clinical outcome was assessed using Harris Hip Score (HHS) and Visual Analogue Scale (VAS). Follow up X-rays were analyzed for signs like collapse of head, decreased joint space, increasing radio opacity, fractures and progressive osteoarthritis.

The data was stored and analyzed in the Statistical Package of the Social Sciences version 22.0. Point estimate at 95% Confidence Interval was calculated along with frequency and proportion for binary data.

## RESULTS

Out of 916 patients who underwent hip surgeries in our hospital, single stage simultaneous bilateral core decompression for bilateral ONFH was done in 12 (1.31%) (0.57-2.04 at 95% Confidence Interval). Among the 12 patients, 8 (66.67%) were males four (33.33%) were female. The mean age of the patients was 36.64±8.93.

**Figure 1.**
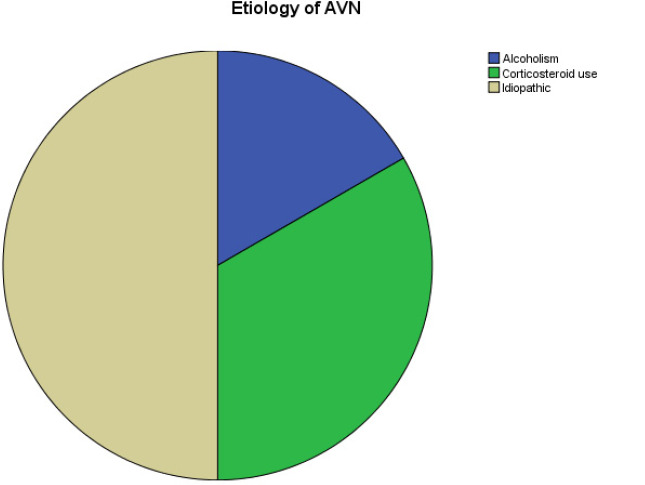
Pie Chart showing the aetiology of ONFH.

Out of the 12 patients, the cause for ONFH was idiopathic in six (50%) followed by prolonged corticosteroid use in four (33.33%) (Figure 1).

Of the 24 hips of the 12 patients, 12 (50%) were in Ficat stage IIa (Table 1).

**Table 1:** Ficat Staging of Osteonecrosis of Femoral Head.

Modified Ficat's staging	No of hips n (%)
I	6 (25)
IIa	12 (50)
IIb	6 (25)
Total	24 (100)

There were originally 12 patients in our study of bilateral ONFH treated with simultaneous core decompression but one patient was lost in the follow-up and excluded from the study leaving 11 patients with 22 hips. There was one (4.5%) hip with superficial wound infection in the immediate postoperative period. However, with daily wound care and antibiotics the infection settled down. None of the hips had fracture neck of femur, injury to lateral cutaneous nerve, excessive bleeding or episodes of thromboembolic phenomenon. Twelve (54.5%) of the hips had good outcomes according to HHS at the end of final follow up at one year. One (4.5%) had poor outcome (Table 2).

**Table 2:** Final Harris Hip Outcome at the end of one year.

Final outcome	Frequency n (%)
Excellent (90-100)	2 (9.1)
Good (80-90)	12 (54.5)
Fair (70-80)	7 (31.8)
Poor (<70)	1 (4.5)
Total	22 (100)

Radiological progression with increase in Ficat Stage was seen in five (22.73%) hips. One (4.55%) case showed progression from Ficat Stage IIa to IIb in 3 months and further progression to stage III at six months. The hip was converted to THA at the end of one year after deterioration in HHS and VAS. Remaining four (18.18%) hips progressed by at least one Ficat stage at final follow up as shown in (Table 3).

**Table 3:** Radiological progression during follow ups.

Radiological complications	One month n (%)	Three month n (%)	Six month n (%)	12 month n (%)
Collapse	0 (0)	0 (0)	1 (4.5)	1 (4.5)
Crescent sign	0 (0)	2 (9.1)	2 (9.1)	4 (18.2)
None	22 (100)	20 (90.9)	19 (86.3)	17 (77.2)
Total	22 (100.0)	22 (100.0)	22 (100.0)	22 (100.0)

**Figure 2.**
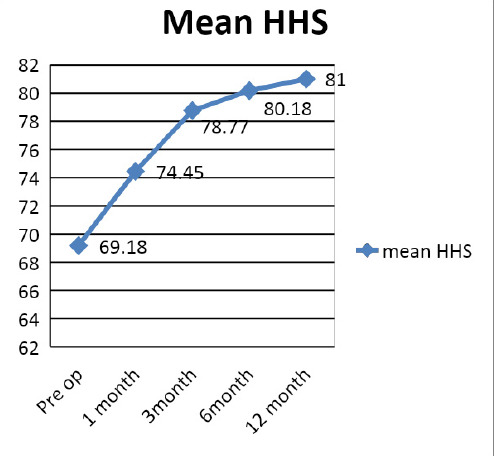
Mean HHS after treatment.

**Figure 3.**
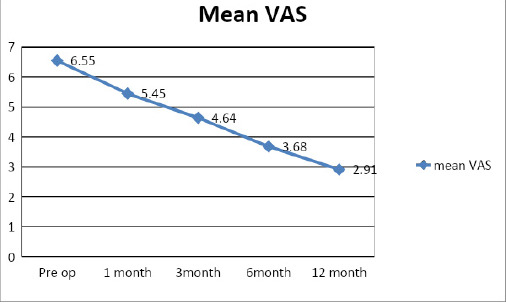
Mean VAS after treatment.

The mean preoperative HHS was 69.18±4.04 and the final HHS of 81±9.05 (Figure 2). Similarly, the mean preoperative VAS was 6.55±0.74 and the final VAS 2.9±2 (Figure 3).

## DISCUSSION

Various hip preserving surgeries have also been investigated for those symptomatic cases of ONFH. A simple core decompression with or without nonvascularized or vascularized bone grafting fibular impaction grafting, core decompression rotational osteotomy and stem cells implantation are some of the commonly done procedures. Core decompression with or without bone grafting is one of the more commonly done procedures. In our study, most patients were in the age group 31-40 (41.7%) and the overwhelming majority were male (66.67%). The data suggests that almost all the patients were male and from the younger age group. Similar findings were presented in a study conducted by Shah et al. in 2015.^6^ Their study included 75% of the patients from age group 20-40 and 80% of them were male.

In our study prolonged corticosteroid usage was the next most common cause of ONFH next to idiopathic. Many of the studies as the one conducted by Shannon and Trousdale^7^ have highlighted the long-term corticosteroid use as an important risk factor. A very common association between a corticosteroid therapy and ONFH was outlined in another study by Adrian C. Fairbank et al.^4^

Modified Ficat and Arlet classification for ONFH was used in our study to classify the hips. Stage I had minor osteopenia and diagnosed with clinico-radiological evaluation. Stage II hips had sclerotic or cystic changes. Stage II was further classified into Stage IIa with focal radiological changes and Stage IIb with crescent sign without flattening of the femoral head. Stage III had flattening of femoral head or femoral head collapse with intact joint space. Similarly, Stage IV had femoral head collapse and osteoarthritis of the hip. Stages III and IV were excluded from the study as several authors have advocated the use of core decompression for pre collapse stage.^3,4^

One of the primary concerns of simultaneous core decompression is having high complication rate mainly the fracture neck of femur followed by others like infection and thromboembolic phenomenon. We have shown a very low complication rate with one hip having postoperative superficial skin infection (4.5%) and no cases of fracture neck of femur. Some of the studies as those done by Camp and Cowell (1986)^8^ reported a 10% incidence of postoperative proximal femoral fracture in 40 cored hips and Hopson and Siverhus (1988)^9^ reported one fracture in 17 patients. Fairbank A et al.^4^ at John Hopkins University School of Medicine had four fractures in 38 patients (22 by simultaneous coring) treated by bilateral core decompression.

The clinical outcome in our study was assessed using HHS and VAS. The preoperative values were compared sequentially till the end of follow up at one year. 9.1% of the patients treated had excellent results and 54.5% had good outcomes according to the final functional evaluation using HHS. The final mean HHS improved to 81 from 69.18. There was considerable improvement in the mean VAS score 6.55 to 2.9. Israelite C et al.^10^ in their retrospective study of 193 patients with 152 bilateral procedures evaluated the change in HHS along with other parameters. They concluded that when bilateral core decompression is indicated, it can be done simultaneously on both hips, allowing earlier treatment of the contralateral hip without risk of increased complications and possibly with a better outcome.

In our study the clinical success rates (no reoperation) after simultaneous bilateral core decompression was 95.45% at the end of one year. Feng W et al.^2^ in their retrospective study from January 2008 to December 2013 used HHS and VAS scores for clinical evaluation along with a series of X-ray images and concluded that one stage hip preserving surgeries for the management of bilateral ONFH could obtain good medium- and long-term outcomes.

Though most studies have indicated that surgical outcome is better than conservative measure in early stages of the disease, some authors have reported little difference.^11^ Authors like Hsu JE et al.^12^ have concluded that core decompression for asymptomatic ONFH is unpredictable. And asymptomatic ONFH lesions particularly in the setting of bilateral disease should be closely observed and surgery reserved for when symptoms arise.

There are a few limitations to our study. Firstly, it was a retrospective study done on a limited sample size. We need more multicentre studies with longer follow ups. Large number of cases might provide better insights. Secondly, the postoperative radiological evaluation was mainly focused on the X-ray images. Postoperative computed tomography (CT) scan and magnetic resonance image (MRI) might provide a more subjective estimation of pathophysiology despite higher financial burden on the patients.

## CONCLUSIONS

We found that a small proportion of hip surgeries were single stage simultaneous bilateral core decompression for osteonecrosis of femoral head in our setting. We believe that core decompression delays the need for total hip replacement in young patients with ischaemic necrosis. Lastly, with a very low complication rate we suggest simultaneous bilateral core decompression a safe procedure. More studies are needed to clarify the difference in outcome between a midterm mean follow up with a range of one year versus a longer term follow up with larger groups of patients in simultaneous bilateral core decompression for ONFH. The procedure requires only a single hospitalization compared to two separate procedures. The ultimate goal is always avoiding arthroplasty in young patients.
